# The Book World of Medicine and Science

**Published:** 1893-05-27

**Authors:** 


### THE BOOK WORLD OF MEDICINE AND SCIENCE.

### BOOK REVIEWS.

TORCH-BEARERS OF HISTORY, a connected series of Historical Sketches. By AMELIA HUTCHISON STIRLING, formerly Lecturer in the Ladies' College, Cheltenham. (T. Nelson and Sons, London, Edinburgh, and New York.)

In this little book Miss Hutchison Stirling has conscientiously striven after simplicity, but it cannot be said that she has succeeded. Simplicity is not gained by merely commencing sentences with "well" and "now," nor can we think that all the torch-bearers are well chosen. Hypatia, though a noble and touching figure, was a heroine of reaction, not of progress, remembered to-day only because of her tragic fate. The work of Homer and Sophocles had, indeed, a historic influence in formulating the myths and legends of old Greece into a religious system, but this is not touched upon; we get only brief sketches of their lives, and the plot of some of their works. Robert the Bruce, with all deference to the author's natural patriotic prejudices, was only a local—one might almost say parochial—hero. It would have mattered little to the world if Scotland and England had been united three centuries earlier. Then why was there not a sketch of Attila, or some of those fierce barbarians who first broke the power of Rome? The Crusades are of immense importance, but was it not Peter the Hermit who should have been taken as their type rather than gallant, foolish, futile Cœur-de-Lion? And why, above all, is there no record of Mahomet, whose blazing torch is not yet near extinction? We cannot think that this book in any way fulfils its promises.

THE DUTIES AND RESPONSIBILITIES OF THE MEDICAL PROFESSION IN RELATION TO THE PUBLIC EYESIGHT. A Presidential Address delivered before the members of the South-Western Branch of the British Medical Association, by LOUIS H. TOSSWILL, B.A., M.B., Cantab, 1892.

It is much to be regretted, though we do not see the remedy, that public opinion takes so long to be influenced in matters of vital importance which are constantly being urged forward into a prominent position by the medical profession; and in no case is this apathy of the public more strongly demonstrated than in the matter of what we may call public eyesight.

The Presidential Address delivered by Dr. Tosswill before the members of the South-Western Branch of the British Medical Association should have a far wider distribution than we fear it will have. Selecting his own specialty for the subject of his address, Dr. Tosswill gives a short *résumé* of the "Duties and Responsibilities of the Medical Profession in Relation to the Public Eyesight," a subject which has recently exercised physicians and surgeons considerably. In the short discourse which Dr. Tosswill delivered, he managed to crowd a large amount of practical common sense, for not only does he emphasise the domestic importance of correcting errors of refraction, but he severely criticises—and we must say not too harshly—the apathy and neglect of our railway companies in not properly and scientifically testing the eyesight and colour-vision of their servants. When we reflect that the proper and safe working of the network of railways of our islands depends on the eyesight of firemen,

THE BOOK WORLD OF MEDICINE AND SCIENCE.—(*continued*).

drivers, and signalmen being perfect, we must see at once that proper inspection and periodic testing is of prime importance for the safety of the public, and that constant agitation and representations to the proper place are the only means by which some protection can be guaranteed to the traveller that he will not be smashed up or killed in accidents.

The increasing number of children that one constantly sees shows that at least to some extent parents are beginning to re-recognise the importance of correcting errors of refraction in their children's eyes. "Many a child," as Dr. Tosswill says, "looked upon as dull or idle or both is simply hypermetropic, and the blame is really due to those who have not supplied him with suitable glasses." And we venture to think that a good many grown up people require education on this matter, and should be made to understand that the ordinary "occulist," *i.e.*, spectaclemaker, is about as incapable for judging the spectacles required as the grocer is who supplies them with so-called patent medicines.

We cannot too strongly commend to the notice of parents the remarks found in the address on the importance of ascertaining the state of a boy's vision before selecting and educating him for whatever profession they or he may choose. It is a frequent occurrence for the physicians attached to our public schools, where special classes are held for army and navy, to have boys brought to them whose eyesight is so defective that it would be quite impossible for them to pass the Medical Board. But there are more, as the Director-General of the Army Medical Department states, having gone through all the training and education required who are rejected for admission to Sandhurst for defective vision or colour blindness.

We fear that there is only too much truth in Dr. Tosswill's quotation, "the boss wouldn't keep me if he saw I had to use spectacles." Employers of labour, especially those requiring skilled workmen, need to be taught that errors of refraction are of very common occurrence, and that a man will work better for his master and with less damage to his own sight if deficiencies in the workman's eyes are properly corrected by the use of spectacles, instead of believing, as they now almost universally do, that a man in glasses cannot work as well as one without.

The same remarks apply to astigmatism, and Dr. Tosswill, with the feelings of a paterfamilias addressing a large number of fathers, gives a striking instance of how his six-year-old son was awakened to the beauty of his two sisters by the proper cylindrical glasses being supplied him.

Passing on then from the medical man's domestic responsibilities to his public ones, Dr. Tosswill calls attention to the testing of vision and colour sense adopted or neglected, as the case may be, by our railway companies. We must again express our heartiest approval of Dr. Tosswill's remarks. The principle which the companies seemed to have followed is to wait for accident of some magnitude and then test the eyesight of the surviving servants, a method of investigation which, as a rule, leads to no result on account of the death of the principal agents. "A man," to quote the address, "with really defective sight or defective colour sense is not fit for any of the three responsible posts of fireman, engine driver, or signal man," and when we consider that up to the present date no Government regulations have been issued regarding the eyesight of railway servants, we shall appreciate the risks

THE BOOK WORLD OF MEDICINE AND SCIENCE—(*continued*).

that are run in railway travelling. The railway population, we are told, is over eight hundred millions, and the safety of this multitude is placed in the hands of men whose sight and consequent ability to perform their duties has only been tested by inspectors and superintendents, and not by the medical officers of the line. By men who are utterly ignorant of the eye, and who make no attempt to test the eyes separately among a class of men who are specially liable to get foreign bodies into their eyes.

"When we come to the methods adopted by the various railway companies," says Dr. Tosswili, "for detecting colour blindness, we find even a worse state of things, for on no less than fifteen English railways, half the Irish, and nearly all the Scotch, the examination simply consists in candidates naming coloured objects, though this method of examination is universally condemned by ophthalmic experts as utterly unsatisfactory and fallacious."

Naturally a workman will not report his eyesight as failing, neither will his mates bear witness against him; "and yet how many cases must there be cataract, nerve atrophy, retinal and choroidal diseases, refracture troubles, and a variety of other diseases in which the sight becomes most seriously affected, whilst the eyes look as well as ever to the casual observer."

We hope it may never be our fortune or misfortune to go a journey in a train piloted by a driver, such as Professor MacHardy came across, who, the Professor was certain, "had never in his life seen the funnel of his own engine." We believe, however, that plenty of instances of a similar, if not of as severe a condition, could be found by a not too exhaustive investigation, and it only tends to point out how necessary it is that some State control should be exercised in selecting servants for such responsible posts.

The conclusions which Dr. Tosswill classes under four heads appear to us not only necessary, but urgent; but we fear that unless the agitation is not taken up by some active and prominent member of our legislature, that it will be a considerable time before they will come into effect.

